# (2*E*)-*N*′-[(*E*)-4-Chloro­benzyl­idene]-3-phenyl­prop-2-enohydrazide monohydrate

**DOI:** 10.1107/S160053681003388X

**Published:** 2010-08-28

**Authors:** Samir A. Carvalho, Edson F. da Silva, Carlos A. M. Fraga, Solange M. S. V. Wardell, James L. Wardell, Edward R. T. Tiekink

**Affiliations:** a FioCruz-Fundação Oswaldo Cruz, Instituto de Tecnologia em Fármacos-Farmanguinhos, Rua Sizenando Nabuco, 100, Manguinhos, 21041-250 Rio de Janeiro, RJ, Brazil; b Programa de Pós-Graduação em Química, Instituto de Química, Universidade Federal do Rio de Janeiro, 21949-900 Rio de Janeiro, RJ, Brazil; c Laboratório de Avaliação e Síntese de Substâncias Bioativas, Faculdade de Farmácia, Universidade Federal do Rio de Janeiro, PO Box 68023, 21941-902 Rio de Janeiro, RJ, Brazil; d CHEMSOL, 1 Harcourt Road, Aberdeen AB15 5NY, Scotland; e Centro de Desenvolvimento Tecnológico em Saúde (CDTS), Fundação Oswaldo Cruz (FIOCRUZ), Casa Amarela, Campus de Manguinhos, Av. Brasil 4365, 21040-900 Rio de Janeiro, RJ, Brazil; f Department of Chemistry, University of Malaya, 50603 Kuala Lumpur, Malaysia

## Abstract

The conformation about each of the imine and ethene bonds in the title hydrazide hydrate, C_16_H_13_ClN_2_O·H_2_O, is *E*. The hydrazide mol­ecule is approximately planar (r.m.s. deviation of the 20 non-H atoms = 0.172 Å). The most significant twist occurs about the ethene bond [C—C=C—C = 164.1 (5)°] and the dihedral angle formed between the benzene rings is 5.3 (2)°]. In the crystal, the presence of N—H⋯O_w_ and O—H⋯O_c_ (× 2; w = water and c = carbon­yl) hydrogen bonds leads to a supra­molecular array in the *bc* plane.

## Related literature

For background to the resurgence of tuberculosis; see Bezerra *et al.* (2006 ▶); Chung & Shin (2007 ▶); Naz *et al.* (2006 ▶). For background to the biological activity of *trans*-cinnamic acid derivatives, see: Carvalho *et al.* (2008 ▶). For background to the development of hydrazide derivatives for biological evaluation, see: Carvalho *et al.* (2008 ▶, 2009 ▶).
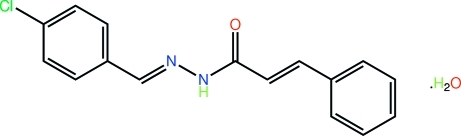


## Experimental

### 

#### Crystal data

C_16_H_13_ClN_2_O·H_2_O
*M* *_r_* = 302.75
Monoclinic, 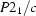

*a* = 34.078 (3) Å
*b* = 5.9824 (6) Å
*c* = 7.2912 (6) Å
β = 95.674 (3)°
*V* = 1479.2 (2) Å^3^
*Z* = 4
Mo *K*α radiation
μ = 0.26 mm^−1^
*T* = 120 K
0.10 × 0.08 × 0.03 mm

#### Data collection

Nonius KappaCCD diffractometer
Absorption correction: multi-scan (*SADABS*; Sheldrick, 2007 ▶) *T*_min_ = 0.492, *T*_max_ = 1.000
8532 measured reflections
2572 independent reflections
2016 reflections with *I* > 2σ(*I*)
*R*_int_ = 0.066

#### Refinement

*R*[*F* ^2^ > 2σ(*F* ^2^)] = 0.084
*wR*(*F* ^2^) = 0.196
*S* = 1.05
2572 reflections
199 parameters
4 restraints
H atoms treated by a mixture of independent and constrained refinement
Δρ_max_ = 0.36 e Å^−3^
Δρ_min_ = −0.37 e Å^−3^

### 

Data collection: *COLLECT* (Hooft, 1998 ▶); cell refinement: *DENZO* (Otwinowski & Minor, 1997 ▶) and *COLLECT*; data reduction: *DENZO* and *COLLECT*; program(s) used to solve structure: *SHELXS97* (Sheldrick, 2008 ▶); program(s) used to refine structure: *SHELXL97* (Sheldrick, 2008 ▶); molecular graphics: *ORTEP-3* (Farrugia, 1997 ▶) and *DIAMOND* (Brandenburg, 2006 ▶); software used to prepare material for publication: *publCIF* (Westrip, 2010 ▶).

## Supplementary Material

Crystal structure: contains datablocks global, I. DOI: 10.1107/S160053681003388X/hb5594sup1.cif

Structure factors: contains datablocks I. DOI: 10.1107/S160053681003388X/hb5594Isup2.hkl

Additional supplementary materials:  crystallographic information; 3D view; checkCIF report

## Figures and Tables

**Table 1:** Hydrogen-bond geometry (Å, °)

*D*—H⋯*A*	*D*—H	H⋯*A*	*D*⋯*A*	*D*—H⋯*A*
N2—H2n⋯O1w	0.86 (2)	1.97 (3)	2.811 (6)	165 (5)
O1w—H1w⋯O1^i^	0.84 (5)	2.05 (5)	2.877 (5)	166 (4)
O1w—H2w⋯O1^ii^	0.85 (4)	2.10 (4)	2.923 (5)	165 (5)

Symmetry codes: (i) 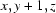; (ii) 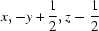.

**Fig. 1.**
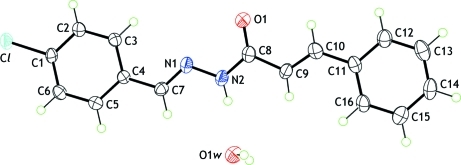
The molecular structure of (I) showing displacement ellipsoids at the 50% probability level.

**Fig. 2.**
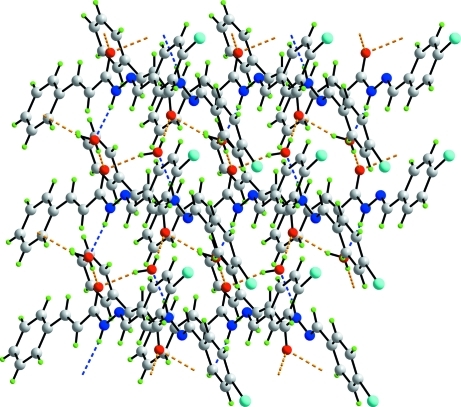
A view in projection down the a axis of the 2-D supramolecular array in the bc plane in (I) with the O–H···O and N–H···O hydrogen bonding shown as orange and blue dashed lines, respectively.

**Fig. 3.**
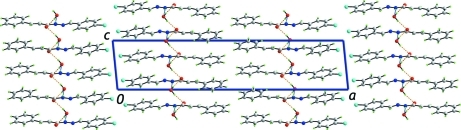
A view in projection down the b axis of the crystal packing in (I) highlighting the stacking of layers. The O–H···O and N–H···O hydrogen bonding shown as orange and blue dashed lines, respectively.

## supplementary crystallographic information

### Comment

Tuberculosis (TB) remains among the world's great public health challenges. Worldwide resurgence of TB is due to two major problems: the AIDS epidemic, which started in the mid-1980's, and the outbreak of multi-drug resistant (MDR) TB (Bezerra *et al.*, 2006; Chung & Shin 2007; Naz *et al.*, 2006). In connection with on-going studies designed to generate novel therapeutic anti-malarial agents, we recently described a new class of isonicotinic and benzoic acid *N*'-(3-phenyl-acryloyl)hydrazide derivatives as attractive anti-tubercular agents (Carvalho *et al.*, 2008). Allied with these investigations are structural studies: the structure of *N*'-[(2*E*)-3-phenylprop-2-enoyl]benzohydrazide was recently reported by us (Carvalho *et al.*, 2009). We have synthesized for biological study a series of PhCH═CHCONHN═CHC_6_H_4_*X* compounds and now we report the crystal and molecular structure of one of these (1: *X* = Cl).

The asymmetric unit of (I) comprises the hydrazide molecule and a water molecule of crystallization. Despite there being twists in the molecule of (I), Fig. 1, the r.m.s. deviation of the 20 non-hydrogen atoms is 0.172 Å [max. and min. deviations = 0.284 (4) for atom N2 and -0.362 (1) Å for the Cl atom]. The dihedral angle formed between the peripheral benzene rings is 5.3 (2) °. The major twist in the molecule occurs about the C9═C10 bond as seen in the value of the C9–C10–C11–C12 torsion angle of 164.1 (5) °. The conformation about the imine [N1═C7 = 1.283 (6) Å] and ethene [C9═C10 = 1.328 (7) Å] bonds is *E* in each case.

The N2–H atom forms a hydrogen bond with the water molecule of crystallization and each O–H forms a hydrogen bond to a symmetry related amide-O, Table 1. The result of the hydrogen bonding is the formation of a supramolecular array in the *bc* plane, Fig. 2, and these stack along the *a* axis, Fig. 3.

### Experimental

The title compound was obtained from the reaction between PhCH═CHC(═ O)NHNH_2_ and 4-chlorobenzaldehyde in ethanol. The mixture was stirred at room temperature for 30 min, when extensive precipitation was observed. The mixture was poured onto cold water and then neutralized with 10% aqueous sodium bicarbonate solution. The sample for X-ray structure determination was grown from its EtOH solution to yield colourless blocks of (I); yield 87%, m.pt. 484.3 K. ^1^H NMR (500.00 MHz, DMSO-d6) δ: 6.72 (1*H*, d, J = 16.0 Hz), 7.44 (3*H*, m), 7.53 (2*H*, d, J = 8.0 Hz), 7.65 (2*H*, m), 7.79 (3*H*, m), 8.06 and 8.25 (1*H*, s, *syn* / anti-*E* isomer), 11.61 and 11.77 (1*H*, s, *syn* / anti-*E* isomer) p.p.m.. ^13^C NMR (125 MHz, DMSO-d6) δ: 116.91, 120.04, 127.67, 128.16, 128.50, 128.64, 128.79, 128.85, 128.92, 129.82, 129.96, 133.04, 133.17, 134.09, 134.36, 134.52, 134.67, 140.65, 141.82, 142.19, 145.27, 161.38, 165.96 p.p.m.

### Refinement

The C-bound H atoms were geometrically placed (C–H = 0.95 Å) and refined as riding with *U*_iso_(H) = 1.2*U*_eq_(C). The O– and N-bound H atoms were located from a difference map and refined with the distance restraint O–H = 0.84 ± 0.01 and N–H = 0.86±0.01 Å, and with *U*_iso_(H) = *zU*_eq_(carrier atom); *z* = 1.5 for O and *z* = 1.2 for N.

### Crystal data

**Table:**

C_16_H_13_ClN_2_O·H_2_O	*F*(000) = 632
*M**_r_* = 302.75	*D*_x_ = 1.359 Mg m^−^^3^
Monoclinic, *P*2_1_/*c*	Mo *K*α radiation, λ = 0.71073 Å
Hall symbol: -P 2ybc	Cell parameters from 19841 reflections
*a* = 34.078 (3) Å	θ = 2.9–27.5°
*b* = 5.9824 (6) Å	µ = 0.26 mm^−^^1^
*c* = 7.2912 (6) Å	*T* = 120 K
β = 95.674 (3)°	Block, colourless
*V* = 1479.2 (2) Å^3^	0.10 × 0.08 × 0.03 mm
*Z* = 4	

### Data collection

**Table:**

Nonius KappaCCD diffractometer	2572 independent reflections
Radiation source: Enraf Nonius FR591 rotating anode	2016 reflections with *I* > 2σ(*I*)
10 cm confocal mirrors	*R*_int_ = 0.066
Detector resolution: 9.091 pixels mm^-1^	θ_max_ = 25.0°, θ_min_ = 3.0°
φ and ω scans	*h* = −40→39
Absorption correction: multi-scan (*SADABS*; Sheldrick, 2007)	*k* = −7→7
*T*_min_ = 0.492, *T*_max_ = 1.000	*l* = −8→8
8532 measured reflections	

### Refinement

**Table:**

Refinement on *F*^2^	Primary atom site location: structure-invariant direct methods
Least-squares matrix: full	Secondary atom site location: difference Fourier map
*R*[*F*^2^ > 2σ(*F*^2^)] = 0.084	Hydrogen site location: inferred from neighbouring sites
*wR*(*F*^2^) = 0.196	H atoms treated by a mixture of independent and constrained refinement
*S* = 1.05	*w* = 1/[σ^2^(*F*_o_^2^) + (0.0421*P*)^2^ + 8.0472*P*] where *P* = (*F*_o_^2^ + 2*F*_c_^2^)/3
2572 reflections	(Δ/σ)_max_ = 0.001
199 parameters	Δρ_max_ = 0.36 e Å^−^^3^
4 restraints	Δρ_min_ = −0.37 e Å^−^^3^

### Special details

**Table:**

Geometry. All s.u.'s (except the s.u. in the dihedral angle between two l.s. planes) are estimated using the full covariance matrix. The cell s.u.'s are taken into account individually in the estimation of s.u.'s in distances, angles and torsion angles; correlations between s.u.'s in cell parameters are only used when they are defined by crystal symmetry. An approximate (isotropic) treatment of cell s.u.'s is used for estimating s.u.'s involving l.s. planes.
Refinement. Refinement of *F*^2^ against ALL reflections. The weighted *R*-factor *wR* and goodness of fit *S* are based on *F*^2^, conventional *R*-factors *R* are based on *F*, with *F* set to zero for negative *F*^2^. The threshold expression of *F*^2^ > 2σ(*F*^2^) is used only for calculating *R*-factors(gt) *etc*. and is not relevant to the choice of reflections for refinement. *R*-factors based on *F*^2^ are statistically about twice as large as those based on *F*, and *R*- factors based on ALL data will be even larger.

### Fractional atomic coordinates and isotropic or equivalent isotropic displacement parameters (Å^2^)

**Table:**

	*x*	*y*	*z*	*U*_iso_*/*U*_eq_	
Cl	0.02800 (3)	−0.2977 (2)	0.18849 (16)	0.0315 (4)	
O1	0.28173 (10)	−0.0171 (6)	0.2229 (5)	0.0330 (9)	
N1	0.20834 (11)	0.1523 (7)	0.1857 (5)	0.0280 (10)	
N2	0.24086 (12)	0.2830 (8)	0.1657 (6)	0.0299 (10)	
H2N	0.2398 (16)	0.420 (3)	0.129 (7)	0.036*	
C1	0.07011 (13)	−0.1339 (9)	0.1827 (6)	0.0240 (11)	
C2	0.10625 (13)	−0.2220 (9)	0.2528 (6)	0.0238 (10)	
H2	0.1074	−0.3662	0.3077	0.029*	
C3	0.14047 (13)	−0.1007 (8)	0.2430 (6)	0.0235 (11)	
H3	0.1652	−0.1615	0.2906	0.028*	
C4	0.13869 (13)	0.1134 (8)	0.1623 (6)	0.0221 (10)	
C5	0.10192 (13)	0.2008 (9)	0.0959 (6)	0.0239 (10)	
H5	0.1004	0.3458	0.0425	0.029*	
C6	0.06758 (13)	0.0787 (9)	0.1071 (6)	0.0267 (11)	
H6	0.0427	0.1401	0.0635	0.032*	
C7	0.17447 (14)	0.2426 (9)	0.1444 (6)	0.0260 (11)	
H7	0.1727	0.3929	0.1023	0.031*	
C8	0.27682 (14)	0.1859 (9)	0.1837 (6)	0.0282 (11)	
C9	0.30969 (14)	0.3385 (9)	0.1550 (6)	0.0290 (11)	
H9	0.3046	0.4928	0.1329	0.035*	
C10	0.34638 (14)	0.2621 (10)	0.1598 (6)	0.0296 (12)	
H10	0.3503	0.1079	0.1869	0.036*	
C11	0.38178 (14)	0.3936 (9)	0.1270 (6)	0.0295 (12)	
C12	0.41917 (14)	0.3067 (10)	0.1809 (7)	0.0339 (12)	
H12	0.4215	0.1632	0.2367	0.041*	
C13	0.45299 (15)	0.4277 (11)	0.1540 (7)	0.0388 (14)	
H13	0.4783	0.3688	0.1935	0.047*	
C14	0.44946 (15)	0.6344 (11)	0.0691 (7)	0.0372 (14)	
H14	0.4725	0.7163	0.0482	0.045*	
C15	0.41234 (15)	0.7235 (10)	0.0140 (7)	0.0342 (13)	
H15	0.4102	0.8662	−0.0433	0.041*	
C16	0.37871 (14)	0.6055 (9)	0.0424 (6)	0.0305 (12)	
H16	0.3535	0.6670	0.0049	0.037*	
O1W	0.23087 (11)	0.6983 (6)	−0.0131 (5)	0.0349 (9)	
H1W	0.2424 (14)	0.797 (8)	0.055 (6)	0.052*	
H2W	0.2444 (13)	0.668 (9)	−0.101 (5)	0.052*	

### Atomic displacement parameters (Å^2^)

**Table:**

	*U*^11^	*U*^22^	*U*^33^	*U*^12^	*U*^13^	*U*^23^
Cl	0.0216 (6)	0.0401 (8)	0.0329 (6)	−0.0052 (6)	0.0028 (5)	0.0042 (6)
O1	0.0311 (19)	0.031 (2)	0.037 (2)	−0.0030 (17)	0.0035 (15)	0.0053 (17)
N1	0.025 (2)	0.036 (3)	0.023 (2)	−0.0076 (19)	0.0022 (16)	0.0007 (19)
N2	0.025 (2)	0.033 (3)	0.032 (2)	−0.006 (2)	0.0062 (17)	0.002 (2)
C1	0.019 (2)	0.033 (3)	0.021 (2)	−0.004 (2)	0.0050 (18)	0.001 (2)
C2	0.025 (2)	0.030 (3)	0.016 (2)	−0.002 (2)	0.0004 (18)	−0.001 (2)
C3	0.022 (2)	0.024 (3)	0.024 (2)	0.002 (2)	0.0024 (19)	−0.004 (2)
C4	0.022 (2)	0.026 (3)	0.019 (2)	0.000 (2)	0.0047 (18)	0.001 (2)
C5	0.028 (2)	0.024 (3)	0.020 (2)	0.003 (2)	0.0014 (18)	−0.004 (2)
C6	0.021 (2)	0.032 (3)	0.028 (3)	0.005 (2)	0.0063 (19)	−0.001 (2)
C7	0.027 (3)	0.025 (3)	0.027 (2)	−0.001 (2)	0.006 (2)	0.001 (2)
C8	0.028 (3)	0.034 (3)	0.022 (2)	−0.005 (2)	0.0005 (19)	−0.004 (2)
C9	0.027 (3)	0.030 (3)	0.029 (2)	−0.008 (2)	0.003 (2)	0.002 (2)
C10	0.029 (3)	0.040 (3)	0.019 (2)	0.000 (2)	−0.0007 (19)	0.001 (2)
C11	0.022 (2)	0.041 (3)	0.025 (2)	0.001 (2)	0.0002 (19)	−0.001 (2)
C12	0.030 (3)	0.043 (3)	0.029 (3)	−0.004 (3)	0.004 (2)	0.000 (3)
C13	0.027 (3)	0.062 (4)	0.027 (3)	0.003 (3)	0.001 (2)	−0.006 (3)
C14	0.027 (3)	0.059 (4)	0.027 (3)	−0.012 (3)	0.006 (2)	−0.002 (3)
C15	0.038 (3)	0.039 (3)	0.027 (3)	−0.008 (3)	0.008 (2)	−0.004 (2)
C16	0.025 (3)	0.043 (3)	0.022 (2)	−0.005 (2)	−0.0036 (19)	0.000 (2)
O1W	0.040 (2)	0.031 (2)	0.034 (2)	−0.0018 (18)	0.0060 (16)	−0.0059 (17)

### Geometric parameters (Å, °)

**Table:**

Cl—C1	1.742 (5)	C8—C9	1.476 (7)
O1—C8	1.256 (6)	C9—C10	1.328 (7)
N1—C7	1.283 (6)	C9—H9	0.9500
N1—N2	1.376 (6)	C10—C11	1.479 (7)
N2—C8	1.351 (6)	C10—H10	0.9500
N2—H2N	0.86 (2)	C11—C12	1.396 (7)
C1—C6	1.385 (7)	C11—C16	1.409 (8)
C1—C2	1.390 (6)	C12—C13	1.392 (7)
C2—C3	1.381 (6)	C12—H12	0.9500
C2—H2	0.9500	C13—C14	1.382 (8)
C3—C4	1.408 (7)	C13—H13	0.9500
C3—H3	0.9500	C14—C15	1.395 (7)
C4—C5	1.399 (6)	C14—H14	0.9500
C4—C7	1.461 (6)	C15—C16	1.379 (7)
C5—C6	1.389 (7)	C15—H15	0.9500
C5—H5	0.9500	C16—H16	0.9500
C6—H6	0.9500	O1W—H1W	0.84 (5)
C7—H7	0.9500	O1W—H2W	0.85 (4)
			
C7—N1—N2	116.8 (4)	N2—C8—C9	114.5 (5)
C8—N2—N1	118.5 (4)	C10—C9—C8	120.6 (5)
C8—N2—H2N	117 (4)	C10—C9—H9	119.7
N1—N2—H2N	124 (4)	C8—C9—H9	119.7
C6—C1—C2	120.8 (4)	C9—C10—C11	126.4 (5)
C6—C1—Cl	120.5 (4)	C9—C10—H10	116.8
C2—C1—Cl	118.7 (4)	C11—C10—H10	116.8
C3—C2—C1	120.2 (5)	C12—C11—C16	119.0 (5)
C3—C2—H2	119.9	C12—C11—C10	119.5 (5)
C1—C2—H2	119.9	C16—C11—C10	121.5 (4)
C2—C3—C4	119.8 (4)	C13—C12—C11	120.8 (6)
C2—C3—H3	120.1	C13—C12—H12	119.6
C4—C3—H3	120.1	C11—C12—H12	119.6
C5—C4—C3	119.0 (4)	C14—C13—C12	119.5 (5)
C5—C4—C7	119.9 (4)	C14—C13—H13	120.3
C3—C4—C7	121.1 (4)	C12—C13—H13	120.3
C6—C5—C4	120.9 (5)	C13—C14—C15	120.5 (5)
C6—C5—H5	119.5	C13—C14—H14	119.8
C4—C5—H5	119.5	C15—C14—H14	119.8
C1—C6—C5	119.1 (4)	C16—C15—C14	120.3 (5)
C1—C6—H6	120.4	C16—C15—H15	119.9
C5—C6—H6	120.4	C14—C15—H15	119.9
N1—C7—C4	119.7 (5)	C15—C16—C11	120.0 (5)
N1—C7—H7	120.1	C15—C16—H16	120.0
C4—C7—H7	120.1	C11—C16—H16	120.0
O1—C8—N2	122.5 (5)	H1W—O1W—H2W	110 (3)
O1—C8—C9	123.0 (5)		
			
C7—N1—N2—C8	−171.2 (4)	N1—N2—C8—C9	178.4 (4)
C6—C1—C2—C3	−1.9 (7)	O1—C8—C9—C10	3.8 (7)
Cl—C1—C2—C3	177.1 (3)	N2—C8—C9—C10	−176.7 (4)
C1—C2—C3—C4	0.2 (7)	C8—C9—C10—C11	177.7 (4)
C2—C3—C4—C5	1.0 (6)	C9—C10—C11—C12	164.1 (5)
C2—C3—C4—C7	−177.9 (4)	C9—C10—C11—C16	−15.9 (8)
C3—C4—C5—C6	−0.7 (6)	C16—C11—C12—C13	0.8 (7)
C7—C4—C5—C6	178.3 (4)	C10—C11—C12—C13	−179.2 (4)
C2—C1—C6—C5	2.3 (7)	C11—C12—C13—C14	−1.4 (8)
Cl—C1—C6—C5	−176.7 (3)	C12—C13—C14—C15	1.3 (8)
C4—C5—C6—C1	−1.0 (7)	C13—C14—C15—C16	−0.5 (8)
N2—N1—C7—C4	180.0 (4)	C14—C15—C16—C11	−0.1 (7)
C5—C4—C7—N1	−172.0 (4)	C12—C11—C16—C15	0.0 (7)
C3—C4—C7—N1	7.0 (7)	C10—C11—C16—C15	180.0 (4)
N1—N2—C8—O1	−2.0 (7)		

### Hydrogen-bond geometry (Å, °)

**Table:**

*D*—H···*A*	*D*—H	H···*A*	*D*···*A*	*D*—H···*A*
N2—H2n···O1w	0.86 (2)	1.97 (3)	2.811 (6)	165 (5)
O1w—H1w···O1^i^	0.84 (5)	2.05 (5)	2.877 (5)	166 (4)
O1w—H2w···O1^ii^	0.85 (4)	2.10 (4)	2.923 (5)	165 (5)

Symmetry codes:  (i) *x*, *y*+1, *z*; (ii) *x*, −*y*+1/2, *z*−1/2.
